# CSF Proteomic Alzheimer’s Disease-Predictive Subtypes in Cognitively Intact Amyloid Negative Individuals

**DOI:** 10.3390/proteomes9030036

**Published:** 2021-08-02

**Authors:** Betty Marije Tijms, Johan Gobom, Charlotte Teunissen, Valerija Dobricic, Magda Tsolaki, Frans Verhey, Julius Popp, Pablo Martinez-Lage, Rik Vandenberghe, Alberto Lleó, José Luís Molinuévo, Sebastiaan Engelborghs, Yvonne Freund-Levi, Lutz Froelich, Lars Bertram, Simon Lovestone, Johannes Streffer, Stephanie Vos, ADNI, Kaj Blennow, Philip Scheltens, Henrik Zetterberg, Pieter Jelle Visser

**Affiliations:** 1 Alzheimer Center Amsterdam, Department of Neurology, Amsterdam Neuroscience, Amsterdam UMC, 1007 MB Amsterdam, The Netherlands; p.scheltens@amsterdamumc.nl (P.S.); pj.visser@amsterdamumc.nl (P.J.V.); 2 Clinical Neurochemistry Laboratory, Sahlgrenska University Hospital, 413 45 Mölndal, Sweden; johan.gobom@neuro.gu.se (J.G.); kaj.blennow@neuro.gu.se (K.B.); henrik.zetterberg@clinchem.gu.se (H.Z.); 3 Department of Psychiatry and Neurochemistry, Institute of Neuroscience and Physiology, Sahlgrenska Academy at the University of Gothenburg, 413 45 Mölndal, Sweden; 4 Neurochemistry Laboratory, Department of Clinical Chemistry, Amsterdam University Medical Centers (AUMC), Amsterdam Neuroscience, 1007 MB Amsterdam, The Netherlands; c.teunissen@amsterdamumc.nl; 5 Lübeck Interdisciplinary Platform for Genome Analytics, University of Lübeck, 23562 Lübeck, Germany; valerija.dobricic@uni-luebeck.de (V.D.); lars.bertram@uni-luebeck.de (L.B.); 6 1st Department of Neurology, AHEPA University Hospital, Makedonia, 546 21 Thessaloniki, Greece; tsolakim1@gmail.com; 7 Alzheimer Center Limburg, School for Mental Health and Neuroscience, Maastricht University, 6211 LK Maastricht, The Netherlands; f.verhey@maastrichtuniversity.nl (F.V.); s.vos@maastrichtuniversity.nl (S.V.); 8 Old Age Psychiatry, University Hospital Lausanne, 1011 Lausanne, Switzerland; julius.popp@chuv.ch; 9 Department of Geriatric Psychiatry, University Hospital of Psychiatry and University of Zürich, 8008 Zürich, Switzerland; 10 Fundación CITA-Alzhéimer Fundazioa, 20009 San Sebastian, Spain; pmlage@cita-alzheimer.org; 11 Neurology Service, University Hospitals Leuven, 3000 Leuven, Belgium; rik.vandenberghe@uzleuven.be; 12 Laboratory for Cognitive Neurology, Department of Neurosciences, KU Leuven, 3000 Leuven, Belgium; 13 IIB-Sant Pau, Hospital de la Santa Creu i Sant Pau, Universitat Autonoma de Barcelona, 08041 Barcelona, Spain; alleo@santpau.cat; 14 Barcelonaβeta Brain Research Center (BBRC), Pasqual Maragall Foundation, 08005 Barcelona, Spain; jlmolinuevo@barcelonabeta.org; 15 Alzheimer’s Disease Unit and Other Cognitive Disorders Unit, Hospital Clinic de Barcelona, 08041 Barcelona, Spain; 16 Reference Center for Biological Markers of Dementia (BIODEM), Institute Born-Bunge, University of Antwerp, 2610 Antwerpen, Belgium; sebastiaan.engelborghs@uzbrussel.be (S.E.); johannes.streffer@acimmune.com (J.S.); 17 Department of Neurology, Universitair Ziekenhuis Brussel and Center for Neurosciences, Vrije Universiteit Brussel, 1090 Brussels, Belgium; 18 School of Medical Sciences, Örebro University, 702 81 Örebro, Sweden; yvonne.freund-levi@ki.se; 19 Center for Alzheimer Research, Division of Clinical Geriatrics, Department of Neurobiology, Care Sciences and Society, Karolinska Institutet, 171 77 Stockholm, Sweden; 20 Department of Geriatric Psychiatry, Zentralinstitut für Seelische Gesundheit, University of Heidelberg, 68159 Mannheim, Germany; lutz.froelich@zi-mannheim.de; 21 Center for Lifespan Changes in Brain and Cognition, Department of Psychology, University of Oslo, 0373 Oslo, Norway; 22 University of Oxford, Oxford OX1 2JD, UK; slovesto@its.jnj.com; 23 AC Immune SA, 1024 Lausanne, Switzerland; 24 Department of Neurodegenerative Disease, UCL Institute of Neurology, London WC1N 3BG, UK; 25 UK Dementia Research Institute at UCL, London WC1E 6BT, UK

**Keywords:** Alzheimer’s disease, cerebrospinal fluid proteomics, risk factors, cognitive functioning, amyloid beta, tau

## Abstract

We recently discovered three distinct pathophysiological subtypes in Alzheimer’s disease (AD) using cerebrospinal fluid (CSF) proteomics: one with neuronal hyperplasticity, a second with innate immune system activation, and a third subtype with blood–brain barrier dysfunction. It remains unclear whether AD proteomic subtype profiles are a consequence of amyloid aggregation, or might exist upstream from aggregated amyloid. We studied this question in 127 older individuals with intact cognition and normal AD biomarkers in two independent cohorts (EMIF-AD MBD and ADNI). We clustered 705 proteins measured in CSF that were previously related to AD. We identified in these cognitively intact individuals without AD pathology three subtypes: two subtypes were seen in both cohorts (n = 49 with neuronal hyperplasticity and n = 44 with blood–brain barrier dysfunction), and one only in ADNI (n = 12 with innate immune activation). The proteins specific for these subtypes strongly overlapped with AD subtype protein profiles (overlap coefficients 92%–71%). Longitudinal p_181_-tau and amyloid β 1–42 (Aβ42) CSF analysis showed that in the hyperplasticity subtype p_181_-tau increased (β = 2.6 pg/mL per year, *p* = 0.01) and Aβ42 decreased over time (β = −4.4 pg/mL per year, *p* = 0.03), in the innate immune activation subtype p_181_-tau increased (β = 3.1 pg/mL per year, *p* = 0.01) while in the blood–brain barrier dysfunction subtype Aβ42 decreased (β = −3.7 pg/mL per year, *p* = 0.009). These findings suggest that AD proteomic subtypes might already manifest in cognitively normal individuals and may predispose for AD before amyloid has reached abnormal levels.

## 1. Introduction

Alzheimer’s disease (AD) is a neurodegenerative disorder and the most common cause of dementia. The pathological hallmarks are aggregation of amyloid in plaques and aggregation of tau in neurofibrillary tangles in the brain and biomarkers for amyloid and tau pathology are now used for the diagnosis of AD [1,2,3,4,5,6]. Recent proteomics studies in plaques, tangles, and cortical tissue suggest that individuals with AD show considerable variability in terms of other pathophysiological processes involved [7,8,9,10]. However, it remains unclear whether such processes are a downstream consequence of amyloid aggregation, or whether they might be dysregulated upstream from amyloid aggregation. Currently the field is starting to test therapies that prevent amyloid aggregation. For example, the A3 trial will test whether inhibiting beta-secretase 1 (BACE1), which is an enzyme that initiates amyloidogenic processing of the amyloid-precursor protein (APP) [11], may prevent amyloid aggregation in older individuals with normal cognition and normal AD biomarkers (https://clinicaltrials.gov (accessed on 20 May 2021)). For this reason, it is important to increase the understanding of interindividual differences in pathophysiological processes that contribute to disease heterogeneity in Alzheimer’s disease.

Cerebrospinal fluid (CSF) contains thousands of proteins, and their concentrations may reflect alterations in ongoing (patho-)physiological processes in vivo. For example, amyloid and tau CSF levels can be used as a biomarker for the presence of AD pathology, which can already be detected in very early stages of the disease when cognition is still normal [12,13,14,15,16]. Furthermore, in cognitively intact older individuals without AD pathology, higher CSF levels of proteins associated with abnormal APP processing predict future amyloid aggregation [15]. This suggests that CSF protein levels may indicate that AD pathophysiological processes have started before aggregated amyloid can be detected. In CSF it is also possible to tease out disease heterogeneity in AD, as we previously identified AD subtypes that show distinct CSF proteomic profiles [17]. One subtype showed hyperplasticity, increased BACE1 activity and high levels of tau, the second showed innate immune system activation and the third subtype showed blood–brain barrier dysfunction, mostly normal tau levels and hypoplasticity. It could be hypothesized that if AD proteomic subtypes exist upstream from amyloid aggregation, it may be possible to identify these cognitively intact older individuals, and that if these processes are specific for AD they should relate to future amyloid and/or p_181_-tau aggregation.

Here we studied this question in cognitively intact individuals with normal AD biomarkers by data-driven cluster analysis of CSF protein levels. We found that two subtypes in cognitively intact individuals with normal AD biomarkers strongly overlapped with the subtypes observed in AD individuals. In a subset of individuals with longitudinal AD biomarkers we found that in these subtypes AD biomarkers in CSF became more abnormal over time. This suggests that distinct AD subtypes may precede amyloid abnormality and may indicate that there could be distinct pathophysiological processes leading to AD.

## 2. Materials and Methods

### 2.1. Participant Description

We selected individuals with intact cognition and normal CSF amyloid β 1–42 (Aβ42) and t-tau measures with available proteomics data from two independent multicenter AD studies, the European Medical Information Framework for Alzheimer’s disease Multimodal Biomarker Discovery study (EMIF-AD MBD [18]) and the Alzheimer’s disease Neuroimaging Initiative (ADNI, adni.loni.usc.edu). Both cohorts included individuals with intact cognition, mild cognitive impairment (MCI), or AD-type dementia as determined according to international consensus criteria [19,20,21,22]. ADNI started in 2003 as a public-private collaboration under the supervision of Principle Investigator Michael W. Weiner, MD. The primary goal of ADNI is to study whether serial magnetic resonance imaging (MRI), positron emission tomography (PET), other biological markers, and clinical and neuropsychological measures can be combined to measure the progression of mild cognitive impairment (MCI) and early Alzheimer’s disease (AD). Please see www.adni-info.org for the latest information. ADNI data was downloaded on 30 March 2018. The institutional review boards of all participating institutions approved the procedures for this study. Written informed consent was obtained from all participants or surrogates.

### 2.2. Cerebrospinal Fluid Data

CSF samples were obtained as previously described [18,23,24]. CSF Aβ42, t-tau, and p_181_-tau levels were measured with INNOTEST ELISAs in EMIF-AD MBD, and in ADNI with the multiplex xMAP Luminex platform (Luminex Corp, Austin, TX, USA) with the INNOBIA AlzBio3 kit (Fujirebio, Ghent, Belgium) at the ADNI Biomarker Core laboratory at the University of Pennsylvania Medical Center. For ADNI biomarker abnormality was defined by Aβ42 levels <192 pg/mL and t-tau levels >93 pg/mL [18,23,24]. In EMIF-AD MBD cut-offs for *p* were study specific as previously reported [17,18,23,24]. Cluster analyses were performed on proteomic data performed using tandem mass tag (TMT) technique with 10 + 1 plexing in EMIF-AD MBD using high-pH reverse phase HPLC for peptide prefractionation [17,25,26]. The EMIF-AD MBD mass spectrometry proteomics data have been deposited to the ProteomeXchange Consortium via the PRIDE [27] partner repository with the dataset identifier PXD019910 and 10.6019/PXD019910. Normalized abundances with associated clinical data can be requested from the EMIF-AD MBD consortium [17]. In ADNI, 4 proteins included were determined with ELISAs, 311 protein fragments determined with Multi Reaction Monitoring (MRM) targeted mass spectroscopy, and 83 proteins measured with Rules Based Medicine (RBM) multiplex. Information on protein assessment and quality control is described at http://adni.loni.usc.edu/data-samples/biospecimen-data/ (accessed on 14 July 2020). For ADNI MRM we used the quality controlled finalized ‘Normalized Intensity’ data [28] (please see for detailed explanation of the normalization procedure the “Biomarkers Consortium CSF Proteomics MRM data set” in the “Data Primer” document at adni.loni.ucla.edu). All proteins (EMIF-AD MBD and ADNI) and protein fragments (ADNI) values were first normalized according to mean and standard deviation values of the control group. Next, for ADNI, protein fragments from MRM measurements were combined into a protein score when these correlated with r > 0.5, and fragments that did not correlate were left out for the present analyses. Eleven proteins were measured by different platforms in ADNI, for which values were averaged if they correlated with r > 0.5 and else we selected the protein as measured by MRM (mean r = 0.74; min r = −0.50, max r = 0.92; for one protein RBM was excluded, another protein (CST3) showed a strong anticorrelation between RBM and MRM of r= −0.85, and was excluded). Only proteins that were observed in 100% of the sample, and that we previously associated with AD in our previous study [17] were considered for subsequent analyses, resulting in total 556 proteins in EMIF-AD MBD and 149 proteins in ADNI (see Supplementary Table S1). A subset of individuals had additional protein measurements available, which we excluded from clustering to use as independent outcomes for subtype interpretation. In ADNI these were Aβ 1–40 and Aβ 1–38 measured with 2D-UPLC tandem mass spectrometry, BACE1 activity, and Elisa measures of neurogranin, neurofilament light, VILIP, YKL40, SNAP25, and sTREM2. In EMIF-AD MBD Elisa measurements were available for Aβ 1–40, Aβ 1–38, neurogranin, neurofilament light, and YKL-40 [18].

### 2.3. APOE e4 Genotyping

ADNI samples were genotyped using either the Illumina 2.5-M array (a byproduct of the ADNI whole-genome sequencing sample) or the Illumina OmniQuad array [29] *APOE* genotype was assessed with two SNPs (rs429358, rs7412) that define the epsilon 2, 3, and 4 alleles, using DNA extracted by Cogenics from a 3 mL aliquot of EDTA blood. In EMIF, *APOE* genotypes were measured using genome-wide SNP genotyping with Global Screening Array (Illumina Inc., San Diego, CA, USA) [29].

### 2.4. Cluster Analyses with Non-Negative Matrix Factorization

We clustered proteins that we previously associated with AD [17] (Supplementary Table S1) using non-negative matrix factorization (NMF). NMF is a dual clustering approach that is based on decomposition of the data by parts, which reduces the dimensionality of data protein expression levels into fewer components which we consider protein profiles [30], and concurrently grouping together individuals into subtypes based on how well their protein expression levels match the protein profiles. NMF is able to capture non-linear patterns associated with a certain subtype, which is an advantage over other correlation-based approaches. We determined for each protein which subtype group showed the highest average levels, and labelled proteins as belonging to a particular subtype accordingly. We used the R package NMF for clustering, with the ‘nonsmooth’ option that ensures sparse cluster solutions with enhanced separability [31]. Person classification to a subtype can vary from run to run because NMF is stochastic. Therefore, we used the co-phonetic coefficient with values ranging from 0 (i.e., unstable solution) to 1 (i.e., subjects are always classified the same) assess subtype classification stability over 50 different runs of NMF. We tested up to 5 clusters, and the optimal number of clusters was determined as the number of clusters for which: 1. The cophonetic correlation was high; 2. Fit compared to a lower cluster number solution was improved at least 2-fold over a random solution; and 3. Silhouette width of the cluster solution was ≥0.5. Clustering analyses were performed separately for each cohort. We used the NMF predict function to label individuals according to the protein cluster that best corresponded with their proteomic expression profile [32]. We performed pathway enrichment analysis for proteins that were characteristic for each subtype using the online Panther application (release 20210224) [33]. We used the ENCODE and ChEA consensus transcription factor database in the Enrichr webserver [34,35] to identify potential upstream drivers of subtype specific protein alterations. We selected pathways that were most consistently associated with the subtypes for visualization, and report all observed pathways in the Supplementary Materials. To determine specificity of proteins for particular cell types we used the BRAIN RNASeq database (http://www.brainrnaseq.org (accessed on 18 November 2018) [36]. Proteins were labelled as being specifically produced by a certain cell type when levels were higher than 50% of the total produced across cell types, as non-specific when none of the cell types was higher than 50%, or as not detected when levels were all <0.2.

### 2.5. Post-Hoc Subtype Comparisons Statistical Procedures

After subtyping, we first quantified consistency with AD subtypes by computing the overlap coefficient of subtype proteomic difference profiles within controls to that of corresponding AD subtypes. The overlap coefficient [37] is the number of overlapping proteins divided by the smallest total protein set size, with 0 indicating no overlap, and 1 indicating that a protein set is a complete (sub)set of the other. We also computed the overlap coefficient to quantify consistency with AD subtypes for the GO biological pathways enriched. Next, we studied whether control subtypes showed changes over time in Aβ42 and p_181_-tau levels, in a subset of ADNI who had repeated measures available. We also studied, if subtypes would show worsening in delayed memory test scores on the ADAS-Cog delayed word recall subscale, since this measure was most sensitive to decline in a previous study [15] (available in ADNI only). Next, we performed post-hoc subtype comparisons on the following characteristics: the proportion of females and APOE e4 carriers, age, CSF levels of t-tau, p_181_-tau, and other established AD CSF markers that were not included in the cluster analyses to provide further independent interpretation of the cluster solutions. All continuous variables (except for age) were standardized according the mean and standard deviation of the control group. Subtype comparisons were performed with general linear models in case of continuous variables with two-sided testing, and with chi square tests for discrete variables. We used the R package ‘emmeans’ to obtain estimated marginalized means. All analyses were performed in R v4.0.3 ‘Bunny-Wunnies freak out’.

## 3. Results

### 3.1. Sample Description

We included 127 controls with intact cognition and normal CSF Aβ42 and t-tau levels (Table 1). Individuals in the EMIF-AD MBD cohort were younger than those in ADNI, and had a lower education, MMSE score, and a higher proportion of *APOE* ε4 carriers.

### 3.2. Three CSF Proteomic Subtypes

**Figure 1:**
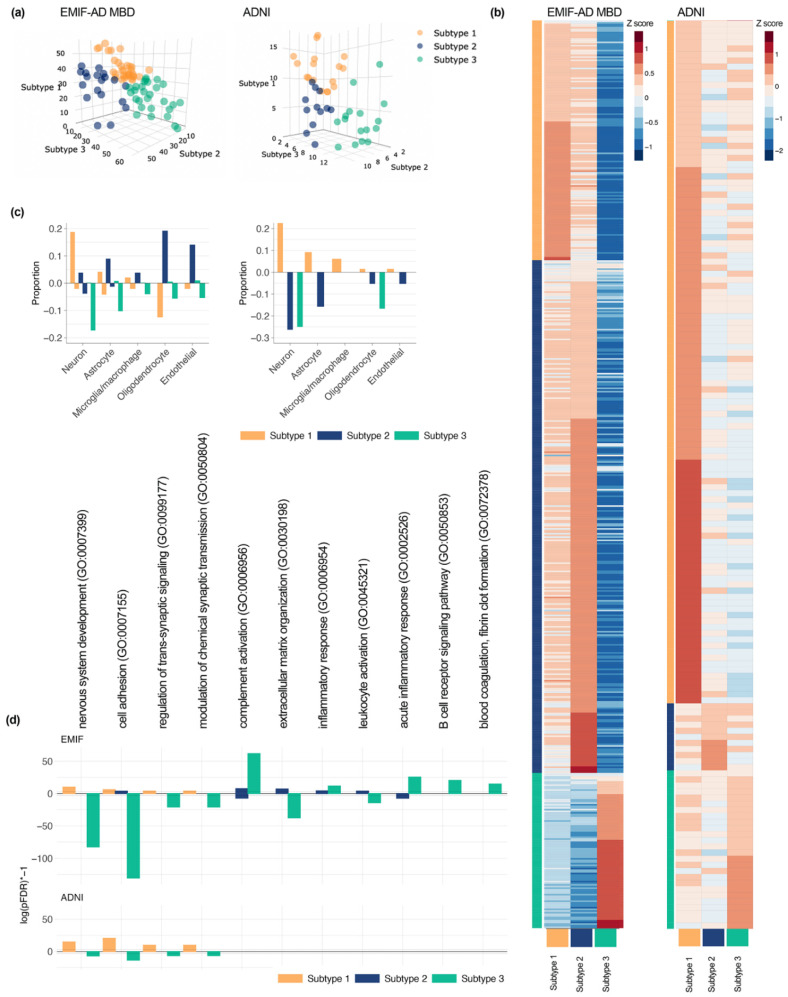
(**a**) Subject scores which reflect how well they match each of the three subtypes, individuals were assigned to the subtype on which they showed the highest loading; (**b**) protein levels averaged across individuals for each subtype (see Supplementary Table S5a,b for statistics of protein level comparisons between subtypes); (**c**) proportion of subtype-specific proteins that were labelled to be specific for a particular cell type (left: EMIF-AD MBD; right: ADNI); (**d**) selection of pathways enriched for subtype-specific proteins (see Supplemental Table S6 for complete list of enriched pathways). For (**c**,**d**): bars going up represent pathways associated with increased proteins, bars going down represent pathways associated with decreased proteins, and in (**d**) absolute numbers represent log(pFDR) * −1, * is times.

Three clusters best described the data of the cognitively intact individuals with normal AD biomarkers for both EMIF-AD MBD and ADNI (Supplementary Table S2). We repeated clustering in the EMIF-AD MBD cohort after excluding three individuals who showed outlying values in their cluster loadings, since these may affect generalizability of the results (see Supplementary Table S3 for outlier characteristics). Three clusters remained the optimal solution, and further analyses were performed on this subset. Subject clustering is shown in Figure 1. In EMIF-AD MBD 32 (41%) and in ADNI 17 (38%) individuals were labelled as subtype 1, 19 (24%) individuals in EMIF-AD MBD and 12 (27%) in ADNI were labelled as subtype 2, and 28 (35%) individuals in EMIF-AD MBD and 16 (36%) in ADNI were labelled as subtype 3. Next, we studied to what extent the control subtypes corresponded to subtypes we previously identified in individuals with abnormal AD biomarkers, by computing consistency of subtype proteomic difference profiles with corresponding AD subtype proteomic difference profiles. We found mostly higher protein concentrations of cognitively intact individuals with normal AD biomarkers subtype 1 compared to 3, which was consistent with increases observed in the neuronal hyperplasticity compared to the blood–brain barrier dysfunction subtypes in AD (overlap scores of 0.98 in EMIF-AD MBD, and 0.97 in ADNI; (Supplementary Tables S4 and S5a,b). Protein increases of subtype 2 compared to subtype 3 were also highly consistent with protein increases we observed in the innate immune activation subtype compared to the blood–brain barrier dysfunction subtype in AD (overlap score of 0.98 in EMIF-AD MBD and of 0.91 in ADNI). Overlap in protein increases of subtype 1 compared to subtype 2 was similar to protein increases observed in the AD hyperplasticity subtype compared to the innate immune activation subtype in ADNI (overlap score of 0.83), but only weakly consistent in EMIF-AD MBD (overlap score of 0.21).

Like the AD neuronal hyperplasticity subtype, hyperplasticity subtype 1 in cognitively intact individuals with normal AD biomarkers showed largely higher than average concentrations of proteins (147 out of 556 in EMIF-AD MBD; 112 out of 149 in ADNI; Supplementary Table S5a,b). In EMIF-AD MBD 22 proteins were significantly higher than both subtype 2 and 3, and thus these proteins were considered to be subtype 1 specific. In ADNI 65 proteins were significantly higher in subtype 1 than subtype 2 and 3. The majority of these proteins were specifically produced by neurons in both cohorts. Almost all pathways associated with specific increased proteins in cognitively intact individuals with normal AD biomarkers and subtype 1 were previously associated with the AD neuronal hyperplasticity subtype (92% EMIF-AD MBD, 90% ADNI). These included nervous system development, cell adhesion, regulation of transsynaptic signaling, and modulation of chemical synaptic transmission. Next, we searched for potential drivers of subtype 1 specially increased proteins, which converged on REST in both cohorts (EMIF-AD MBD *p_adjusted_* = 0.02; ADNI *p_adjusted_* = 1.21 × 10^−8^, Supplementary Table S7), which was also found in subtype 1 individuals with AD.

In subtype 3, proteins that were increased in subtype 1 were *decreased*, which was similar to the decreases observed in the AD blood–brain barrier dysfunction subtype. Subtype 3 showed largely lower than average concentrations of proteins (456 out of 556 in EMIF-AD MBD; 105 out of 145 in ADNI). Of these, 424 proteins in EMIF-AD MBD and 18 proteins in ADNI were significantly lower than both subtype 1 and 2. The majority of these proteins were produced by neurons and astrocytes. A large percentage of the pathways associated with subtype 3 specifically decreased proteins were also previously associated with decreased proteins in the blood–brain barrier dysfunction subtype in AD (76% EMIF-AD MBD, 87% ADNI). Pathways enriched consistently in both cohorts and previously in the AD blood–brain barrier dysfunction subtype were nervous system development, cell adhesion, regulation of transsynaptic signaling, and modulation of chemical synaptic transmission, which were also associated with subtype 1 specifically increased proteins. Potential drivers of protein decreases in this subtype was REST in both cohorts (EMIF-AD MBD *p_adjusted_* = 2.08 × 10^−13^; ADNI *p_adjusted_* = 0.02), similar as in the AD blood–brain barrier dysfunction subtype. Control subtype 3 further showed specific increases for 87 proteins in EMIF-AD MBD, and 2 in ADNI. Thirty-five (40%) of these specifically increased proteins were previously associated with blood–brain barrier dysfunction (Supplementary Table S5a) [38]. Pathway analyses for these proteins from EMIF-AD MBD showed 89% overlap with those we previously associated with increased proteins in the AD blood–brain barrier dysfunction subtype, including acute inflammatory response, B cell receptor signaling pathway, and blood coagulation fibrin cloth formation. No transcription factors were associated with subtype 3 specifically increased proteins.

Subtype 2 showed mostly higher than average protein concentrations in EMIF-AD MBD (457 out of 556 proteins), but in ADNI most proteins had lower than average concentrations (125 out of 149 proteins). Of increased proteins, 89 proteins were significantly different from subtypes 1 and 3 in EMIF-AD MBD, and 18 in ADNI. The majority of increased proteins in EMIF-AD MBD subtype 2 individuals were produced by oligodendrocytes, and endothelial cells. No pathways were enriched for ADNI subtype 2 specific proteins, and no specific cell type involvement was observed. In EMIF-AD MBD, pathways associated with subtype 2 specifically increased proteins overlapped 71% with pathways previously associated with the innate immune system subtype, and included complement activation, extracellular matrix organization, inflammatory response, and leukocyte activation, in line with the AD immune activation subtype. No transcription factors were associated with proteins specifically increased in control subtype 2.

### 3.3. Longitudinal Comparisons of CSF Proteomic Subtypes on Amyloid and p-Tau Levels, and Delayed Memory Functioning

We next tested whether the subtypes differed in their risk to develop AD pathology, by estimating changes over time in CSF Aβ42 and p_181_-tau levels, which was available in ADNI only (n = 45, mean ± SD 3.2 ± 1.2 repeated samples over mean ± SD 3 ± 1.9 years). Subtype 1 individuals showed decreases in Aβ42 towards abnormal levels (β ± SE = −4.4 ± 1.9 pg/mL per year; *p* = 0.03) and increases in p_181_-tau towards abnormal levels (β ± SE = 2.6 ± 0.9 pg/mL per year; *p* = 0.01). Subtype 2 individuals showed increases in p_181_-tau (β ± SE = −3.1 ± 1.1 pg/mL per year; *p* = 0.01) and no changes in Aβ42 levels (β ± SE = −3.7 ± 2.32 pg/mL per year, *p* = 0.12). Subtype 3 individuals showed decreases towards more abnormal levels in aβ42 (β ± SE = −5.6 ± 2.0 pg/mL per year; *p* = 0.009), but no changes in p_181_-tau levels (β ± SE = 1.29 ± 1.0 pg/mL per year, *p* = 0.22). Comparing delayed memory scores at baseline showed similar performance between subtypes (*p* = 0.83). Repeated delayed memory test scores over showed worsening over time in subtype 3 individuals (β ± SE = 0.20 ± 0.07, *p* = 0.005), and no significant changes in subtype 1 and 2 individuals (subtype 1: β ± SE = 0.06 ± 0.06, *p* = 0.33; subtype 2: β ± SE= 0.15 ± 0.08; *p* = 0.08). Differences in slopes between subtypes were not significant (p_interaction_ = 0.30).

### 3.4. CSF Proteomic Subtypes Comparisons on Other Biological Characteristics

**Figure 2:**
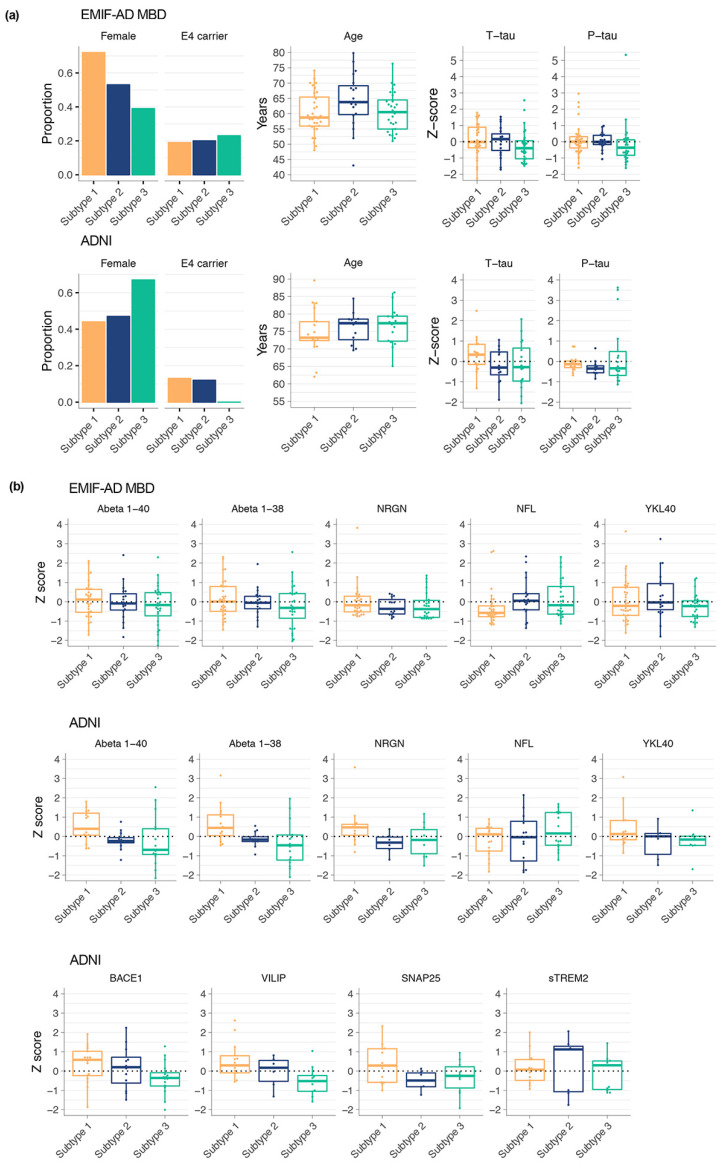
Subtype comparisons on biological characteristics. (**a**) Comparison of subtypes for the proportion of females and APOE e4 carriers, as well as age, t-tau, and p-tau levels; (**b**) comparisons of subtypes on other CSF markers that were not included in clustering (see Supplemental Table S8 for statistics).

Finally, we compared subtypes on other biological characteristics. In EMIF-AD MBD and in ADNI no differences were found amongst subtypes in the proportion of *APOE* ε4 carriers or on average age (Figure 2; Supplementary Table S8). Subtype 1 individuals showed a higher proportion of females than subtype 3 (*p* = 0.02) in EMIF-AD MBD, while no sex differences were found in ADNI (all *p* > 0.05). In ADNI, subtype 1 individuals showed higher levels of BACE1 activation compared to subtype 3 (*p* = 0.03; Figure 2b), and higher levels of aβ40 and aβ38 compared with subtype 2 and 3 (aβ40: 1 vs. 2 *p* = 0.0495; 1 vs. 3 *p* = 0.02; aβ38: 1 vs. 2 *p* = 0.04; 1 vs. 3 *p* = 0.004). In both EMIF-AD MBD and in ADNI, subtype 1 showed highest levels of t-tau, and subtype 3 the lowest, although these differences were not significant (all *p* > 0.05). Subtype 1 in ADNI showed higher levels of VILIP (*p* = 0.009), neurogranin (*p* = 0.046), and CH3L1 (*p* = 0.03) and tended to show higher levels of SNAP-25 (*p* = 0.08). These differences in CSF markers were similar, although attenuated, as we previously observed between AD subtypes 1 (neuronal hyperplasticity) and 3 (blood–brain barrier dysfunction).

## 4. Discussion

The main finding of this study is that CSF proteomic profiles for AD pathophysiological subtypes may already be present in older individuals with intact cognition and a normal AD biomarker profile. Specifically, we identified a subgroup of cognitively intact individuals with evidence for a neuronal hyperplasticity, and another subgroup with evidence for blood–brain barrier dysfunction, both of which we previously observed in AD. Only these two subtypes also showed increased risk for amyloid aggregation over time. Furthermore, the neuronal hyperexcitation subtype also showed increases in p_181_-tau levels over time, while the blood–brain barrier dysfunction subtype did not. Another subtype in controls did not show clear correspondence with an AD subtype, and did not show changes in amyloid over time. Our findings suggest that alterations in processes related to neuronal hyperplasticity or blood–brain barrier dysfunction may exist before Aβ 1–42 and p_181_-tau have become abnormal in CSF. However, the possibility that these subgroups may reflect normal physiological variability between individuals cannot be excluded. Still, the presence of blood–brain barrier dysfunction in subtype 2, and the elevated tau levels in subtype 1 suggest that these proteomic patterns reflect, possibly in part, pathophysiological processes. Future studies should further clarify this issue by collecting repeated CSF proteomics and amyloid and tau measures in cognitively normal older individuals with an initially normal AD CSF profile.

Studies so far demonstrated the usefulness of CSF proteomic analyses to capture disease heterogeneity in AD [17,39,40]. We now show that some of the AD subtype specific processes may already be detected before amyloid aggregation in older individuals with intact cognition and AD biomarker values. Our finding that protein increases in CSF related to APP metabolism precede amyloid aggregation in subtype 1 is in line with observations from other studies [15,41], suggesting that increased amyloid production may play a role in sporadic AD, but only for a specific subgroup of individuals. It must be noted, however, that repeated memory test scores over time for subtype 1 individuals in ADNI did not show decline, and so it is unclear whether these processes would directly impact on cognitive function. An important implication of our findings is that amyloid prevention trials that target BACE1 activity, may only be beneficial for this group of individuals. This subtype further also showed increased levels of a group of proteins related to neuronal plasticity processes. These proteins were associated with the transcriptional repressor factor REST, which is an important regulator of neuronal development and plasticity related processes [42,43,44]. A previous iPSC model from sporadic AD patients showed similar increases in proteins related to neuronal development, which also converged on a role for REST [44]. Those neurons showed increased excitability, amyloid and tau secretion. It is unclear why REST is lost during aging, but possibly reduced integrity of nuclear lamina may lead to translocation of REST from the nucleus into the cytosol [44].

The presence of a subtype indicative of blood–brain barrier dysfunction in cognitively intact individuals with normal AD biomarkers, may indicate an alternative route towards AD pathology [45]. These individuals showed specific increases in proteins that have been associated with blood–brain barrier permeability [38]. Blood-derived proteins such as albumin, immunoglobins, and prothrombin, which we observed to be increased in this subtype have been associated with pericyte loss [46]. Such damage may lead to further buildup of aggregated proteins through hampered clearance, but also may invoke an inflammation response. Possibly, blood–brain barrier permeability is compromised by very early changes in amyloid damaging the vasculature [46]. With aging the blood–brain barrier becomes more permeable, and this might contribute to cognitive decline [46,47,48,49]. In our analyses, individuals with this subtype were the only ones to show decline on delayed memory test scores over time. Blood–brain barrier dysfunction could lead to decreased perfusion and impaired nutrient delivery to the brain, which may contribute to pathophysiological responses of the brain [46,49]. The mainly decreased concentrations of proteins of this subtype overlapped largely with those increased in the neuronal hyperplasticity subtype, which also converged on REST. Since protein levels were decreased, it might be that this subtype shows overexpression of REST, resulting in hypoplasticity. REST overexpression has been reported in ischemic conditions [42,50]. Possibly, blood–brain barrier dysfunction may lead to hypoxia that increases REST expression. It would be of interest to measure REST levels in brain tissue of individuals who present with this subtype. Proteins decreased in this subtype also showed specific enrichment for processes related to autophagy and chaperone mediated autophagy (in EMIF-AD MBD only). Dysfunctional autophagy is a well-established process in AD, with dystrophic neurites showing mostly a buildup of autophagic vacuoles, in addition to aggregated tau and microtubule proteins [51]. Decline in autophagic processing is observed with aging [52] and may lead to intracellular aggregation of amyloid, as well as decreased amyloid secretion [53]. A recent study reported that mice lacking lysosome-associated membrane protein 2A, which is an isoform of the LAMP2 gene that participates in chaperone mediated autophagy, showed increased intracellular aggregation of a large number of proteins including PDIA, PPIA, and PARK7, which we also observed decreased in our CSF proteomic data in this subtype [54]. It could be hypothesized that both REST overactivation or dysfunctional autophagy related processes will lead to further decreases in CSF protein levels, and future studies should investigate this by measuring proteomics in repeated CSF samples over time.

Finally, in both EMIF-AD MBD and in ADNI, we observed another subtype that showed a less consistent correspondence across cohorts with subtypes we previously identified in AD. Although there was some overlap in the pathways associated with this subtype in EMIF-AD MBD with those we previously observed in the AD innate immune activation subtype, this cohort’s relative subtype differences did not overlap with those observed in AD. For ADNI the proteomic profile of this subtype showed a better correspondence with the AD innate immune system activation subtype, but no pathways were enriched and so could not be compared. This may mean that a proteomic profile associated with innate immune activation is down-stream from amyloid aggregation in AD. Alternatively, since in this study these individuals did not show decreases in amyloid over time, perhaps, this subtype could reflect normal individuals.

We found CSF proteomic subtypes in control cases that resembled those we previously observed in AD. Still, a potential limitation of the present study is that we only had repeated CSF and cognition over time available for the ADNI cohort, and so we are unable to verify whether the subtypes we identified in EMIF-AD MBD would show similar changes in amyloid and tau over time as in ADNI. Future studies should further investigate this question by collecting repeated proteomic, amyloid, and tau samplings over time, as well as cognitive tests. Another limitation is that although the total group of individuals we studied was large, the different subgroup sizes were small, making it more difficult to detect subgroup differences. Furthermore, it might be that more subgroups exist that are related to development of AD, and larger initial group sizes are required in order to be able to capture such subgroups if they exist. Finally, our study was cross-sectional, and although two subtypes showed highly consistent proteomic differences to those observed in AD, repeated proteomic sampling over time is required to further verify whether their proteomic profiles become more like those observed in AD.

## 5. Conclusions

Proteomic AD subtypes can already be detected in cognitively normal individuals. These subtype profiles might represent pathophysiological changes upstream from amyloid and/or p-tau aggregation. These results show that CSF proteomics may have use in identifying subtype specific early changes in AD.

## Figures and Tables

**Table 1:** Study participant characteristics.

Characteristic	EMIF-AD MBD (n = 82)	ADNI (n = 45)
Age in years, mean (SD)	61.1 (7)	75.8 (6) *
Female, n (%)	47 (57)	23 (51)
Years of education, mean (SD)	11.9 (3.5)	15.6 (3) *
MMSE, mean (SD)	28.6 (1.3)	29.2 (0.6) *
≥1 *APOE* ε4 allele, n (%)	14 (22)	4 (8) *
Amyloid β 1–42, mean (SD) ‘	0 (1)	247.5 (29.2)
P_181_-tau, mean (SD) ‘	0 (1)	20.3 (9.4)
T-tau, mean (SD) ‘	0 (1)	57.1 (13.1)

‘ Variables were Z transformed in EMIF-AD MBD based on control values in order to harmonize across centers. * Differs between EMIF-AD MBD and ADNI with *p* < 0.05.

## Data Availability

The EMIF-AD MBD mass spectrometry proteomics data have been deposited to the ProteomeXchange Consortium via the PRIDE partner repository with the dataset identifiers PXD019910 and 10.6019/PXD019910.

## Supplementary Materials

The following are available online at https://www.mdpi.com/article/10.3390/proteomes9030036/s1, Table S1: Average (SD) protein levels for AD groups, Table S2: Fit statistics of solutions for increasing number of clusters (2 to 5), Table S3: Descriptive comparison of individuals who were excluded from EMIF-AD MBD due to outlying values on cluster loadings, Table S4: Overlap of AD and controls for proteins that differ between subtypes, Table S5: Average protein levels according to subtypes, comparisons amongst subtypes, and cell type production assignment, (a) in EMIF-AD MBD, (b) in ADNI, Table S6: GO biological pathway enrichment, Table S7: Transcription factor enrichment, Table S8: Proportions or estimated marginal means (se) for biomarker comparisons from Figure 2.
